# Pregnancy related back pain, is it related to aerobic fitness? A longitudinal cohort study

**DOI:** 10.1186/1471-2393-12-30

**Published:** 2012-04-17

**Authors:** Eva Thorell, Per Kristiansson

**Affiliations:** 1 Department of Public Health and Caring Sciences, Family Medicine and Preventive Medicine unit, Uppsala University, Box 564, SE-751 22 Uppsala, Sweden; 2 School of Health and Medical Sciences, Örebro University, SE-702 81 Örebro, Sweden

## Abstract

**Background:**

Low back pain with onset during pregnancy is common and approximately one out of three women have disabling pain. The pathogenesis of the pain condition is uncertain and there is no information on the role of physical fitness. Whether poorer physical conditioning is a cause or effect of back pain is also disputed and information from prospective studies needed.

**Methods:**

A cohort of pregnant women, recruited from maternal health care centers in central Sweden, were examined regarding estimated peak oxygen uptake by cycle ergometer test in early pregnancy, reported physical activity prior to pregnancy, basic characteristics, back pain during pregnancy and back pain postpartum.

**Results:**

Back pain during the current pregnancy was reported by nearly 80% of the women. At the postpartum appointment this prevalence was 40%. No association was displayed between estimated peak oxygen uptake and incidence of back pain during and after pregnancy, adjusted for physical activity, back pain before present pregnancy, previous deliveries, age and weight. A significant inverse association was found between estimated peak oxygen uptake and back pain intensity during pregnancy and a direct association post partum, in a fully adjusted multiple linear regression analysis.

**Conclusions:**

Estimated peak oxygen uptake and reported physical activity in early pregnancy displayed no influence on the onset of subsequent back pain during or after pregnancy, where the time sequence support the hypothesis that poorer physical deconditioning is not a cause but a consequence of the back pain condition. The mechanism for the attenuating effect of increased oxygen uptake on back pain intensity is uncertain.

## Background

Chronic medical conditions are in focus for the development of strategies aimed at improving population health worldwide. This is also true for chronic pain conditions leading to impaired or non-existent ability to exercise, as physical inactivity is associated with development of chronic diseases. Musculoskeletal disorders constitute an estimated 90% of all chronic pain, of which back pain contributes to a high extent.

During pregnancy there is a remarkably increased prevalence of low back pain, as compared with the non-pregnant state. Prevalence rates between 61% and 88% of back pain with onset during current pregnancy are reported, as compared with one-year prevalence of back pain, irrespective of onset, among women of the same age of 40% in the general population. This means that a high proportion of women with previously healthy backs experience onset of back pain in pregnancy. The pregnancy related back pain varies from mild discomfort to severely debilitating pain of several months' duration [1-3]. In most women, pregnancy-related back pain disappears during the first six months after delivery. However, a fairly high proportion still experience low back pain that seriously interferes with daily activities two years after childbirth [4-7].

The cause and pathogenesis of the development and course of pregnancy related low back pain remain uncertain. Known determinants of pregnancy related low back pain are previous pregnancies and deliveries, hormonal contraceptive use before first pregnancy, physically demanding work and emotional distress [8-10]. However, the fraction of explained variance of these factors is small.

Whether physical inactivity causes low back pain or whether low back pain causes patients to decrease their physical activity and become physically weaker is disputed [11]. Smeets et al. [12] reported that patients with moderately to severely disabling low back pain had lower aerobic fitness levels than healthy subjects matched for age, sex and physical activity. As in other studies, a cross-sectional design was used, which means that the timing of the onset of poorer physical condition was unclear. Prospective research on healthy subjects has not identified low activity or fitness levels as significant risk factors for developing chronic low back pain [12]. Physical fitness levels can be assessed by submaximal incremental exercise methods, such as cycle ergometer test, which have shown good agreement with oxygen uptake measured with gas analyses as gold standard [13-15].

Thus a cohort study of women followed from early pregnancy to postpartum was identified in order to study the cause and effect mechanism of physical fitness and development of pregnancy related low back pain. The purpose of the present study was to evaluate the effects of physical fitness in early pregnancy, measured as estimated peak oxygen uptake by cycle ergometer test and reported physical activity prior to pregnancy, on the development and course of back pain during pregnancy and after delivery.

## Methods

### Setting

Female residents of Sweden have the right to attend a maternal health center during pregnancy free of charge. The centers are operated by the county councils, or are subcontracted to the councils. Some of the centers are located at hospitals, others in the community. They are staffed by general practitioners, midwives and administrative staff. They all follow the same general procedure with repeated appointments during pregnancy, and one postpartum.

### Study population

**Figure 1:**
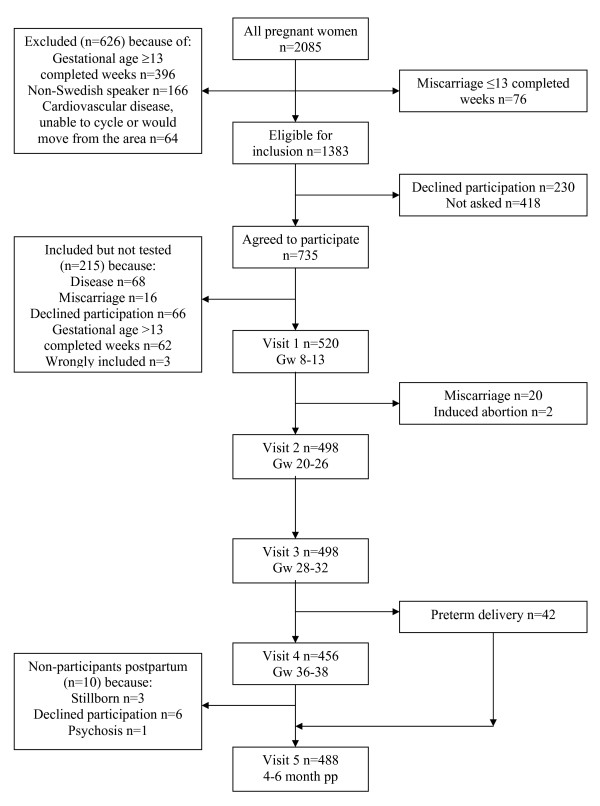
**Study population**. Flow chart of the study population.

Between March 27, 2001 and June 5, 2003, all women in early pregnancy who attended eight maternal health centers in the city of Örebro (population 128,000), and two each in the municipalities Kumla and Hallsberg (populations 20,000 and 15,000) close to Örebro, were identified, a total of 2,085 women. As shown in Figure 1, 1,350 women declined participation in the study or were excluded for various reasons, leaving 735 women who agreed to participate. Of these, 215 did not take the cycle ergometer test, leaving a final study population of 520 women.

Participation rates across the centers ranged from 40% to 70%. Information on non-participating women was retrieved from the medical records of the maternal health centers. None of the main variables used in the study showed any statistically significant association with participation rate.

### Data collection

Data were collected at four appointments, on average at 10.9 (range 5-21), 24.0 (range 18-26), 29.7 (range 27-34), 36.5 (range 35-40) completed gestational weeks, and 21.2 (range 12-54) weeks postpartum. Information was sought about basic physical characteristics, physical activity and oxygen uptake during and after pregnancy.

At the first appointment height was measured without shoes with a wall-mounted tape measure to the nearest centimeter. Weight was measured at all appointments with indoor clothing without shoes on a lever balance, as kilograms to one decimal. The women completed a questionnaire on leisure time physical activity undertaken to maintain or improve fitness or health during the past four weeks at the first and last appointments. The frequency of physical activity was classified as never (= 1), now and then (= 2), once or twice a week (= 3), three to five times a week (= 4), or more than five times a week (= 5). Previous back pain which led to consultation of a physician, physiotherapist, or chiropractor was recorded.

From the second appointment onwards, the location of pain was indicated by the women on a pain sketch. More than one location could be indicated. The back locations were coded as cervical spine, thoracic spine, lumbar spine, lumbosacral spine and sacral spine. In addition, the current back pain intensity was measured on a visual analogue scale (VAS) 100 mm long; 0 mm indicating no pain and 100 mm indicating intolerable pain. Reported pain intensities were attributed to the respectively reported pain locations.

Aerobic capacity was estimated with the submaximal cycle ergometer heart rate method (Monark Exercise Ergometer 828E bicycle, adjustable to individual height), at the first and last appointment, held some time between 8 a.m. and 4.30 p.m., under supervision of the same experienced test administrator (KA). During the test, the women wore light clothing and sport shoes. Room temperature was 18-20°C. The women were instructed not to eat a light meal one hour before or a heavy meal 2-3 h before test, and to avoid strenuous physical activity for one day before test. Nicotine use was not allowed for one hour prior to the test. In case of an on-going infectious disease the test was postponed for two weeks. Heart rate was determined every minute using a wireless chest pulse belt. A pedaling rate of 50 revolutions per minute was kept constant by use of a metronome. Initial workload, 50 W or 75 W, was based on the women's reported physical activity levels, and increased by 25 W per minute until a steady state heart rate of 125·beats or more·per minute was reached, after which the women cycled for at least 6 min until two consecutive heart rates, one minute apart, differed by 3 or fewer beats·per minute. The heart rate values obtained were used to estimate absolute peak oxygen uptake in liters per·minute ($\dot{V}O_{2\text{peak},\;\text{est}\text{.}}$), according to the Åstrand and Ryhming nomogram [16].

The Research Ethics Committee of Örebro University, Sweden, approved the study.

### Statistical analysis

Statistical analyses were performed using the SAS software, version 9.2 (SAS Institute Inc., Cary, NC, USA). Means and proportions were calculated using standard techniques. For the regression analyses data on reported back pain or no back pain from each visit and women's estimates of pain intensity were concatenated to create a data set consisting of pain reports and the corresponding pain intensity estimates throughout pregnancy. Cox regression and General Linear Model were used for regression analysis, and the latter also to produce a model and figure of adjusted back pain intensity by absolute *VO*_*2*peak, est._. No multicollinearity problem was found. Only two-tailed tests were used. Statistical tests were considered significant if p < 0.05.

## Results

Characteristics of the study population are shown in Table 1. Almost half the women reported physical activity at least once a week and the mean absolute $\dot{V}O_{2\text{peak},\;\text{est}\text{.}}$ was 2.4 L·min^-1^. Previous back pain irrespective of pregnancy was reported by nearly half of the women and 8% reported sickness absence because of back pain before the present pregnancy. Back pain of any location at any point during present pregnancy was reported by almost 8 out of 10 women, with lumbosacral and sacral pain as the most commonly reported locations. At the postpartum visit the prevalence of any reported back pain was half or that reported during pregnancy. The mean intensity of back pain increased successively by gestational age to maximum 39 mm on the VAS in late pregnancy, and subsequently declined to 18 mm at 22 weeks postpartum.

**Table 1:** Characteristics of all 520 women included in the study

Characteristic	n	Mean or proportion
Absolute $\dot{V}O_{2\text{peak},\;\text{est}\text{.}}$, early pregnancy (L/min)	520	2.4 (0.5)

Physical activity ≥ once a week (%)	259/520	49.8

Previous back pain, irrespective of pregnancy (%)	238/520	45.8

Sick-leave due to back pain before pregnancy (%)	43/520	8.3

No previous pregnancy (%)	218/520	41.9

No previous delivery (%)	258/520	49.6

Age (yr)	520	29.0 (4.4)

Weight, gestational week 12 (kg)	520	68.1 (12.6)

Height (m)	520	1.67 (0.06)

University education (%)	520	43.9

Current smoker (%)	520	18.5

Back pain location throughout present pregnancy:		

Cervico-thoracic (%)	69/459	15.0

Lumbar (%)	67/459	14.6

Lumbosacral (%)	274/471	58.2

Sacral (%)	253/469	53.9

Any back pain location (%)	373/479	77.9

Back pain of any location postpartum (%)	179/488	36.7

Back pain intensity (mm)		

Gestational week 24	498	33.7 (31.5)

Gestational week 30	497	38.0 (31.3)

Gestational week 36	455	39.2 (32.0)

Post partum week 22	488	18.3 (26.8)

Back pain = cervico-thoracic, lumbar, lumbosacral or sacral pain location. $\dot{V}O_{2\text{peak},\;\text{est}\text{.}}$ = estimated peak oxygen uptake.

The number and proportion of women reporting back pain at each appointment during and after pregnancy are shown in Table 2. During pregnancy the prevalence rates of cervico-thoracic and lumbar pain locations were < 10%, without significant changes postpartum. The corresponding prevalence rates of lumbosacral and sacral pain were stable at about 30-37% during pregnancy and displayed a significant decrease (p < 0.0001) at the postpartum appointment to 24% and 11%, respectively. The proportion of women with more than one back pain location were at the pregnancy visits between 14% and 17% and at the postpartum visit 11%.

**Table 2:** Prevalence of back pain during and after pregnancy by back pain location

		Back pain location											
		
		Cervico- thoracic		Lumbar		Lumbo- sacral		Sacral		More than one location		Any location	
**Time**	**N**	**n**	**%**	**n**	**%**	**n**	**%**	**n**	**%**	**n**	**%**	**n**	**%**

Gw 24	498	30	6	28	6	157	32	152	30	69	14	278	56

Gw 30	497	40	8	33	7	185	37	157	32	86	17	306	62

Gw 36	455	39	8	26	6	161	35	142	31	78	17	263	58

22 w pp	488	52	11	36	7	120	24	53	11	53	11	179	37

Number and proportion of women reporting back pain during and after pregnancy by back pain location.

*Gw *gestational week. *N *total number of women, *n *number of women with the actual back pain location.

### Association between back pain and possible determinants in early pregnancy

Cox regression analysis showed no association between the hazard (incidence) of back pain in any back pain location during pregnancy or back pain location at the postpartum visit on the one hand and absolute $\dot{V}O_{2\text{peak},\;\text{est}\text{.}}$, measured in early pregnancy on the other. However, an inverse significant association was displayed with age and back pain during pregnancy (HR 0.96, p = 0.0005) and back pain during pregnancy and postpartum (HR 0.96, p = 0.0007). Analyses including possible confounding factors did not change these findings.

Possible determinants in early pregnancy of intensity of back pain during pregnancy are shown in Table 3. In the univariate regression analyses absolute $\dot{V}O_{2\text{peak},\;\text{est}\text{.}}$, physical activity, age and university education were significantly inversely associated and previous back pain, previous deliveries, weight and current smoking was significantly positively associated with back pain intensity. In a multiple linear regression analysis all factors remained significant except university education and current smoking. The R^2 ^of the full model was 0.12. A multiple linear regression analysis was similarly performed to find determinants for pain intensity at the postpartum appointment: back pain before pregnancy was significantly and positively associated with pain intensity but neither of the other factors (data not shown). The R^2 ^of that model was 0.07.

**Table 3:** Effects on back pain intensity of factors in early pregnancy in linear regression analyses

	Crude		Adjusted	
**Covariates**	**Estimate**	**p**	**Estimate**	**p**

Absolute $\dot{V}O_{2\text{peak},\;\text{est}\text{.}}$ (L/min)	-6.0	0.0003	-5.2	0.0019

Physical activity	-4.8	< 0.0001	-3.3	< 0.0001

Previous back pain	13.3	< 0.0001	12.6	< 0.0001

Previous delivery	4.5	< 0.0001	4.1	0.0006

Age (yr)	-0.9	< 0.0001	-1.3	< 0.0001

Weight (kg)	0.4	< 0.0001	0.3	< 0.0001

University education	-3.1	< 0.0001	-0.6	0.35

Current smoking	3.4	0.019	-0.6	0.66

$\dot{V}O_{2\text{peak},\;\text{est}\text{.}}$ = estimated peak oxygen uptake.

### Association between back pain intensity and oxygen uptake

**Figure 2:**
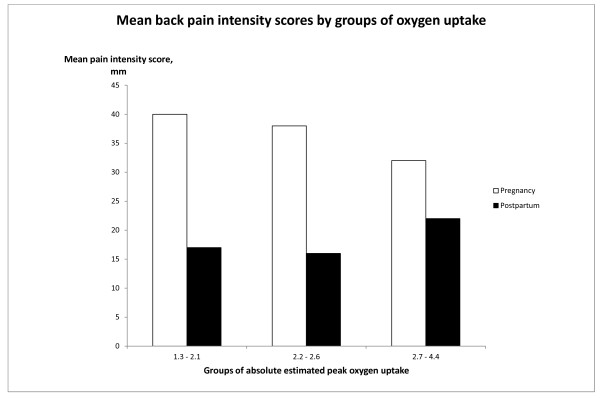
**Outcome measure**. Mean of back pain intensity scores during and after pregnancy, adjusted by determinants in early pregnancy, and presented by groups of estimated absolute oxygen uptake in early pregnancy.

To illustrate the effect of absolute $\dot{V}O_{2\text{peak},\;\text{est}\text{.}}$ on reported back pain intensity, during and after pregnancy, the women were grouped by absolute $\dot{V}O_{2\text{peak},\;\text{est}\text{.}}$ into tertials as, 1.3 to 2.1, 2.2 to 2.6 and 2.7 to 4.4 L/min. The pain intensity scores during pregnancy (adjusted by the independently significant determinants reported physical activity, previous back pain, previous delivery, age and weight), by the absolute $\dot{V}O_{2\text{peak},\;\text{est}\text{.}}$ groups are presented in Figure 2. From the group with the lowest to the group with highest absolute $\dot{V}O_{2\text{peak},\;\text{est}\text{.}}$, the mean back pain intensity scores decreased in a dose-response manner, from 40 mm to 32 mm with significant differences between the group with the lowest absolute $\dot{V}O_{2\text{peak},\;\text{est}\text{.}}$ and the other two groups, p = 0.0003 and p = 0.004, respectively. After delivery the influence of absolute $\dot{V}O_{2\text{peak},\;\text{est}\text{.}}$ was the opposite compared with during pregnancy, but no significant difference was shown.

## Discussion

In this prospective cohort study, oxygen uptake and physical activity in early pregnancy displayed no influence on the onset of back pain during or after pregnancy, while among women reporting pregnancy related back pain physical fitness attenuated the intensity of pregnancy related back pain throughout the pregnancy but not after delivery. This speaks for the hypothesis that poorer physical condition in combination with back pain is not a cause but a consequence of the pain condition.

The strengths of the present study were the prospective approach, use of validated methods and the number of participants who performed the cycle ergometer test. One limitation of the study was the number of non-participants. However, there was no difference in the main variables between participants and non-participants. Hence, it would probably be possible to extrapolate the results of the present study to healthy pregnant women in general. The lack of more precise information on the onset of back pain was another limitation. However, in the present study this was to some extent adjusted for by including reported back pain before the present pregnancy in the multivariate analysis.

To the best of our knowledge, no previous study has investigated the influence of oxygen uptake capacity on development of back pain in subsequent pregnancy and postpartum. However, there are studies of non-pregnant populations with back pain that imply an inverse association between aerobic fitness and back pain [12,17] although these findings are disputed by others [18]. In addition, absolute $\dot{V}O_{2\text{peak},\;\text{est}\text{.}}$ has been inversely correlated with facet degeneration, a significant problem in all chronic low back pain patients although not clearly related to pain [19]. As regards a possible preventive effect of physical activity level on development of pregnancy-related back pain, the results of a retrospective study with its inherent problem regarding recall bias, indicate a decreased frequency of back pain in relation to the amount of regular physical activity before pregnancy [20]. In addition, the cross-sectional methodology used in the above studies made it impossible to evaluate the cause and effect mechanism.

Regular physical exercise reduced pain intensity among pregnant Iranian women but not the frequency of back pain [21]. Lower back pain and neck/shoulder pain has also been found to be inversely associated with regular exercise during pregnancy in a Norwegian study by Owe et al. [22]. This is in accord with the results in the present study where pain intensity was influenced by oxygen uptake and physical activity while the prevalence of back pain was similar to that found in previous studies [2,23-26].

The mechanism underpinning reduced back pain intensity in relation to increased $\dot{V}O_{2\text{peak},\;\text{est}\text{.}}$ and increased physical activity, respectively, can only be speculated about. Firstly, incipient or present pregnancy-related back pain may have reduced the women's ability to perform the ergometer cycle test in early pregnancy. This view is supported by the facts that a higher level of pain before testing is associated with prematurely quitting the bicycle test [12,27] and that maximal exertion in low back pain patients was limited [17]. Secondly, a direct effect of oxygen uptake and fitness on pain perception may be a possibility. Although animal research strongly supports this hypothesis [28] it is disputed in human beings [29,30] and has been shown to only provide temporary relief from pain in healthy individuals [31] and in people with low back pain [32]. Thirdly, there may be co-variation between oxygen uptake and muscular strength, the latter a suggested protective factor for back pain in pregnancy [33,34].

## Conclusions

Oxygen uptake and physical activity in early pregnancy displayed no influence on the onset of subsequent back pain during or after pregnancy, where the time sequence support the hypothesis that poorer physical deconditioning is not a cause but a consequence of the back pain condition. The mechanism for the attenuating effect of increased oxygen uptake on back pain intensity is uncertain.

## Competing interests

The authors declare that they have no competing interests.

## Authors' contributions

ET: conceived the study and participated in its design, coordination and acquisition of data, and contributed to analyses and interpretation of data and drafted the manuscript. PK: conceived the study, contributed to analyses and interpretation, and drafted the manuscript. Both authors read and approved the final manuscript.

## Pre-publication history

The pre-publication history for this paper can be accessed here:

http://www.biomedcentral.com/1471-2393/12/30/prepub
